# Antibiotic resistance indicator genes in biofilm and planktonic microbial communities after wastewater discharge

**DOI:** 10.3389/fmicb.2023.1252870

**Published:** 2023-09-05

**Authors:** Sarah Haenelt, Hans-Hermann Richnow, Jochen A. Müller, Niculina Musat

**Affiliations:** ^1^Department of Isotope Biogeochemistry, Helmholtz Centre for Environmental Research, Leipzig, Germany; ^2^Institute for Biological Interfaces (IBG 5), Karlsruhe Institute of Technology, Eggenstein-Leopoldshafen, Germany; ^3^Department of Biology, Section for Microbiology, Aarhus University, Aarhus, Denmark

**Keywords:** biofilm, surface water, spatiotemporal monitoring, *sul1*, *sul2*, *intI1*, aquatic ecosystem, One Health

## Abstract

The spread of bacteria with antibiotic resistance genes (ARGs) in aquatic ecosystems is of growing concern as this can pose a risk of transmission to humans and animals. While the impact of wastewater treatment plant (WWTP) effluent on ARG abundance in surface waters has been studied extensively, less is known about the fate of ARGs in biofilms. The proximity and dense growth of microorganisms in combination with the accumulation of higher antibiotic concentrations in biofilms might render biofilms a reservoir for ARGs. Seasonal parameters such as water temperature, precipitation, and antibiotic concentrations should be considered as well, as they may further influence the fate of ARGs in aquatic ecosystems. Here we investigated the effect of WWTP effluent on the abundance of the sulfonamide resistance genes *sul1* and *sul2*, and the integrase gene *intI1* in biofilm and surface water compartments of a river in Germany with a gradient of anthropogenic impact using quantitative PCR. Furthermore, we analyzed the bacterial community structure in both compartments via 16S rRNA gene amplicon sequencing, following the river downstream. Additionally, conventional water parameters and sulfonamide concentrations were measured, and seasonal aspects were considered by comparing the fate of ARGs and bacterial community diversity in the surface water compartment between the summer and winter season. Our results show that biofilm compartments near the WWTP had a higher relative abundance of ARGs (up to 4.7%) than surface waters (<2.8%). Sulfonamide resistance genes were more persistent further downstream (>10 km) of the WWTP in the hot and dry summer season than in winter. This finding is likely a consequence of the higher proportion of wastewater and thus wastewater-derived microorganisms in the river during summer periods. We observed distinct bacterial communities and ARG abundance between the biofilm and surface water compartment, but even greater variations when considering seasonal and spatiotemporal parameters. This underscores the need to consider seasonal aspects when studying the fate of ARGs in aquatic ecosystems.

## Introduction

As a step toward limiting the global spread of antimicrobial resistance, it is crucial to remind ourselves that humans, animals, and the environment are interconnected, which has already been well expressed as the basis of the One Health approach (The World Health Organization et al., 2019). The freshwater environment links all terrestrial life, and can therefore be a dissemination route of antibiotic-resistant bacteria and the resistance genes (ARGs) they carry (Berendonk et al., 2015; Larsson and Flach, 2022). The flux of clinically relevant ARGs and the dynamics of their pool sizes in anthropogenically impacted waterbodies are less certain, rendering it important to better understand the mechanisms and factors that influence the fate of these genes in such ecosystems. A key source of ARGs and antibiotics pollution in aquatic ecosystems is untreated and treated wastewater (Proia et al., 2016; Wang et al., 2020). Well-operated wastewater treatment plants (WWTP) are efficient in attenuating concentrations of phosphorous and fixed nitrogen compounds, but they are not specifically designed to remove antibiotic-resistant bacteria, ARGs, and antibiotics (Chaturvedi et al., 2021). Modifying existing or constructing new WWTPs with improved capability for their removal is a substantial task. In the EU, for example, with its comparatively extensive treatment infrastructure, the wastewater of 447 million people is treated in about 26,500 WWTP at investment and operational costs of €39 billion per year (OECD, 2020). Further monitoring of the level of ARG contamination of surface waters will be helpful to guide stakeholders on whether, where, and when to implement improved wastewater treatment.

Cost-effective assessment of ARG contamination can be achieved by quantification of marker genes (Berendonk et al., 2015). The *sul1* and *sul2* genes, which confer resistance to sulfonamides, are among the earliest used markers and most frequently detected ARGs in WWTP effluent (Pruden et al., 2006; Wang et al., 2020). The environmental presence of these ARGs mirrors the common prescription of sulfonamides in human medicine and food animal production, as well as their chemical stability (Martin-Laurent et al., 2019). Likewise, the abundance of the integrase gene *intI1* of the clinical class 1 integron has increased globally in natural environments as a result of anthropogenic activities (Gillings et al., 2008). The genes *intI1* and *sul1* usually show a strong positive correlation in environmental samples, as both co-occur in the classical class 1 integron (Gillings et al., 2015). However, some studies show an independent trend, such as an increase in the relative abundance of *intI1* without a concomitant increase in *sul1* relative abundance (Koczura et al., 2016; Haenelt et al., 2023). While the biological reason for this observation remains to be elucidated, it is an example of how genes associated with antimicrobial resistance that share the same point source can have different environmental fates. Therefore, site monitoring ideally includes the enumeration of several ARGs. In our study, we have chosen *sul1* and *sul2* to assess the antimicrobial resistance status in the Holtemme river, due to their widespread occurrence in aquatic ecosystems globally and their established recognition as indicator genes over many years (Pruden et al., 2006). Furthermore, the prevalence of the *intI1* gene was monitored as a proxy for anthropogenic pollution (Gillings et al., 2015; Yue et al., 2021).

Multiple studies have focused on the fate of ARGs and the effect of anthropogenic pollution on planktonic microorganisms in the surface water or sediment compartment of rivers downstream WWTPs (e.g., Harnisz et al., 2020; Borsetto et al., 2021; Chaturvedi et al., 2021; Hutinel et al., 2021; Pesce et al., 2021; Kasuga et al., 2022). Less attention has been given to the fate of ARGs and the effect of WWTP effluent on bacterial communities in the biofilm compartment of rivers, which we define here as the biological interface between the sediment and the overlaying surface water compartment (Luo et al., 2020). Biofilms are known to play an important role in the functioning of aquatic ecosystems, e.g., in nutrient cycling, transformation of pollutants, and as reservoirs of microorganisms (Flemming et al., 2016). The proximity and dense growth of microorganisms within biofilms, the accumulation of antibiotics resulting in higher concentrations than in the surrounding environment, and their ability to accumulate mobile elements make them a favorable environment for the spread of ARGs (Proia et al., 2016; Flores-Vargas et al., 2021). In addition, sediment samples contain material collected some distance below the surface of the riverbed, where ambient conditions are likely different from those in the biofilm, and hence ARG prevalence could be different in the two compartments.

Furthermore, the effect of seasonal variations on the abundance of *sul1*, *sul2*, and *intI1* in WWTP effluent-impacted streams is currently uncertain. For example, Sabri et al. (2020) reported slightly higher concentrations of these marker genes in a Dutch river during the summer season, while Koczura et al. (2016) found a higher occurrence of the genes in a Polish river during cold months. The latter authors hypothesized that higher antibiotic consumption during colder months might have contributed to the higher ARG concentrations. In contrast, studies targeting the wet and dry seasons in China have not shown significant seasonal differences in the occurrence of ARGs (Chen et al., 2013; Yuan et al., 2014; Mao et al., 2015). Since multiple environmental parameters including water temperature and availability of nutrients vary throughout the year, it is difficult to determine their individual impact. Moreover, the urban discharge fraction, i.e., the proportion of WWTP effluent relative to the total volume of a stream, can be substantially different in summer and winter season, which likely has a strong effect on the spread of ARG. The effect could be exacerbated in regions of the world where climate change is expected to cause more frequent hot and dry summer seasons in the future (Gudmundsson et al., 2021).

This study aimed to assess the effect of wastewater discharge on *sul1*, *sul2*, and *intI1* prevalence comparatively in biofilm and surface water compartments at six sampling sites in a small river along its gradient of anthropogenic impact. We aimed to elucidate the role of the riverine biofilm compartment as a reservoir for ARGs in the aquatic environment, with special emphasis on the effect of wastewater discharge. Furthermore, we investigated the effects of seasonality on the abundance of ARGs and bacterial community diversity in surface waters by comparing results obtained during the current summer sampling campaign with data from a previous study conducted in a winter season (Haenelt et al., 2023).

## Materials and methods

### Study region and sampling sites

This study investigated the Holtemme, a 47 km long river originating in the Harz mountains of Saxony-Anhalt, Germany. The river is considered near-pristine in the mountainous region, while downstream anthropogenic impact increases due to wastewater discharge, rectification, settlements, and widespread farming activities (Inostroza et al., 2016; Karthe et al., 2017; Wollschläger et al., 2017). In the mountainous region, low concentrations of caffeine and the insecticide diethyltoluamide indicate a mild impact by hikers (Weitere et al., 2021). The sediment in the Holtemme is mostly sandy with pebbles and cobbles (Kunz et al., 2017). Five small streams flow into the Holtemme in the study area, all of which contribute little to the water volume of the river. During the sampling period in the summer season of 2022, a stream entering the Holtemme between Site 5 and 6 had dried up completely. For this study, two sampling sites were selected in the “pristine” part of the river, one directly at the discharge point of an activated sludge-based, tertiary WWTP, one about 150 m downstream of the WWTP, and two others 8 and 13 km further downstream. The latter site is at the upstream outskirts of the next city (Halberstadt), which has its own WWTP. All six sampling sites have been previously described in detail (Haenelt et al., 2023).

### Sampling and sample preparation

Planktonic and biofilm samples were collected during a dry period in the summer season of 2022 on five sampling days (August 9th, three consecutive days from August 23rd to 25th, and September 1st) at all six sites. Rain events were rare and the average rainfall was less than 2.3 mm per day (Supplementary Figure 1). Water temperature and pH were measured at all sites using a SenTix41 probe (Xylem Analytics, Germany). NO_3_-N, total phosphorous, and water depth were obtained from the MOBICOS stations located at Sites 1 and 5 (Fink et al., 2020), and physicochemical parameters from Site 3 (WWTP discharge) were kindly provided by the WWTP Silstedt.^1^

Planktonic samples were collected in sterilized and pre-rinsed 1-L glass flasks at an approximate water depth of 5 cm. Biofilm samples were collected with a spatula from the water-facing side of stones covering the riverbed and transferred into tubes containing 1 mL 1xPBS. To obtain a sufficient amount of biomass, approximately 10 cm^2^ of surface area was used for biofilm sampling at Sites 1, 2, 5, and 6. At Sites 3 and 4, which were characterized by dense biofilms, approximately 1 cm^2^ was sampled. All samples were stored in a thermobox with cold packs and transported to the laboratory within 3 h. Immediately upon arrival, biofilm samples were stored at 8°C until DNA extraction within the next 2 days. Planktonic samples were further treated as follows. One liter of river water from each site except Site 3 (500 mL) was filtered using 0.22 μm pore size PSE PALL filters (diameter 47 mm, Pall Corporation, New York, NY, USA) and stored at −20°C until DNA extraction. Additionally, 100 mL flow-through was collected for each site and stored at 8°C overnight before solid phase extraction (SPE) and sulfonamide concentration measurements via HPLC-MS.

### SPE and HPLC-MS analysis

Flow-through was concentrated 100-fold using SPE with Oasis HLB 6cc 500 mg columns (Waters, CT USA) before HPLC-MS was carried out as described in Haenelt et al. (2023). In brief, a calibration curve from 0.01 μg/L to 2 μg/L of a sulfonamide mixture containing sulfamethoxazole (SMX), sulfadiazine (SDZ), and sulfamethazine (SMZ) was generated, and all samples were measured in technical triplicates. HPLC-MS analysis was carried out using a Zorbax Eclipse Plus Rapid Resolution HT-C18 (100 mm × 3 mm, 1.8 μm) column on a 1,260 Infinity II HPLC (Agilent Technologies, Santa Clara, CA, USA) coupled to a QTRAO 6500 + MS/MS (AB Sciex, UK).

### DNA extraction

The biofilm samples were vortexed and centrifuged for 5 min at 13,000 *g* to harvest microorganisms. The supernatant was discarded and the sediment was resuspended in 100 μL BE buffer (Macherey Nagel, Germany). For planktonic samples, filters were cut into small pieces and divided into two parallel 2-mL tubes containing 200 μL BE buffer and approximately 0.15 g of 1-mm zirconium beads (Carl Roth, Germany) each. The individual tubes were agitated for 20 s at 4 m/s on a FastPrep 24 instrument (MP Biomedicals, Germany) and centrifuged in a benchtop centrifuge (neoLab, Germany). The supernatant from parallel tubes was collected and pooled. To maximize the final DNA yield, an additional 100 μL of BE buffer was added to the tubes containing the filter pieces, vortexed, and centrifuged again, and the supernatant was pooled with the previous one. DNA extraction was performed using the NucleoSpin Microbial DNA Kit (Macherey Nagel, Germany) following the manufacturer’s protocol.

### 16S rRNA gene amplicon sequencing

The diversity of bacterial communities in planktonic and biofilm samples was determined for all sites and sampling campaigns by preparing a 16S rRNA gene amplicon library as described (Haenelt et al., 2023). We conducted a nested PCR of the variable region V3 using the primer pairs specified in Supplementary Table 1. Performing a nested PCR was necessary to obtain sufficient DNA yield for the planktonic samples. Amplicons were sequenced with the NextSeq 500/550 High Output Kit v2.5 on an Illumina NextSeq 550 instrument. Bacterial community analysis was performed using QIIME 2 (Bolyen et al., 2019), DADA2 (Callahan et al., 2016), and the taxonomic classifier “Silva 138 99% OTU full-length sequences” (Bokulich et al., 2018; Robeson et al., 2021). Phyloseq (McMurdie and Holmes, 2013) was used for further data analysis in R. The dataset for Site 1 on August 9th was removed due to low sequencing depth. Rarefaction curves can be found in Supplementary Figure 2. For statistical analyses of alpha diversity measurements, we performed Kruskal-Wallis and Dunn’s tests using the package rstatix (Kassambara, 2023). An overview of the statistical significance of differences in alpha diversity, both between sampling sites and within each sampling site, comparing planktonic and biofilm samples, can be found in Supplementary Table 2. To compare microbial beta diversity in biofilm and planktonic samples, we applied non-metric multidimensional scaling (NMDS) of Bray-Curtis dissimilarities using the vegan package (Oksanen et al., 2022). To assess seasonal influences on bacterial community structure and diversity in the surface water compartment, we did NMDS with data obtained for planktonic samples in this study and data from our previous study from the 2020/2021 winter season (Haenelt et al., 2023). PERMANOVA tests were performed to evaluate the statistical significance of differences in beta diversity between sampling sites, within each sampling site comparing both surface water and biofilm compartments (Supplementary Table 3), as well as between the summer and winter season.

### Quantitative PCR

Absolute abundances of *sul1*, *sul2*, *intI1*, and the 16S rRNA gene were determined with SYBR Green-based quantitative real-time PCR using established primers (Supplementary Table 1) and a protocol from Haenelt et al. (2023). For planktonic samples, copy numbers (CN) were normalized to CN/100 mL river water. For biofilm samples, CN were normalized to CN/sample and are therefore not directly comparable with each other. Relative abundances of *sul1*, *sul2*, and *intI1* were calculated by dividing their absolute abundances by those of the 16S rRNA gene. The statistical significance of differences in absolute and relative ARG abundances between sampling sites and seasons was determined using the package rstatix (Kassambara, 2023). We performed statistical analyses employing Kruskal-Wallis and Dunn’s tests using the package rstatix (Kassambara, 2023). Results for the relative abundance of the target genes, encompassing comparisons of both sampling sites and riverine compartments within each sampling site can be found in Supplementary Table 4. Additionally, we compared the abundance of all four target genes quantified in the current study with data acquired during a winter season in our previous study (Haenelt et al., 2023). We aimed to identify notable differences in both absolute and relative abundance between the two seasons for each sampling site using Dunn’s test, and the outcome of these analyses is depicted in Supplementary Table 5.

## Results and discussion

### Physicochemical parameters

**FIGURE 1:**
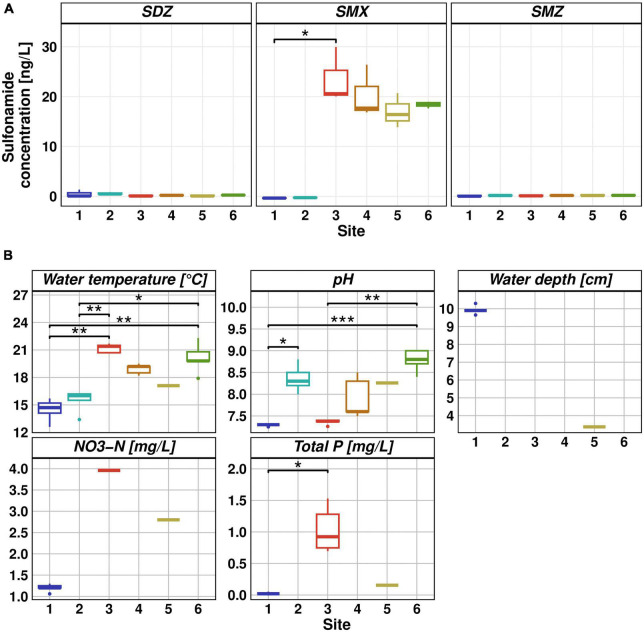
**(A)** Sulfonamide concentrations measured via HPLC-MS. Measurement was done in five replicates and sulfadiazine (left), sulfamethoxazole (middle) and sulfamethazine (right) concentrations are reported as box whisker plots for each site. **(B)** Physicochemical parameters. Measurements were obtained from MOBICOS monitoring stations (Site 1 and 5, https://www.ufz.de/index.php?en=39611), WWTP Silstedt (Site 3, https://www.wahb.eu/) and manually using a SenTix41 probe (Site 2, 4, and 6). From left to right and top to bottom: water temperature [°C], pH, water depth [cm], NO_3_-N [mg/L], and total P [mg/L]. Significant differences between sampling sites are represented by asterisks (Dunn’s test, * ≤ 0.05, ** ≤ 0.01, *** ≤ 0.001).

Our monitoring study showed that WWTP effluent was the primary source of SMX, nitrogen, and phosphorus pollution. Downstream of the discharge point, our data show decreasing SMX as well as nutrient concentrations. The highest SMX concentration was found at Site 3, with a median concentration of 20 ng/L, resulting in 17.7 ng/L SMX at Site 4. This finding is in line with previous studies on the Holtemme (Krauss et al., 2019; Beckers et al., 2020; Švara et al., 2021) and with numerous publications which showed that WWTP effluent is the primary source of antibiotic pollution in aquatic ecosystems (Carvalho and Santos, 2016; Rodriguez-Mozaz et al., 2020; Zhuang et al., 2021; Larsson and Flach, 2022). Sulfonamides are typically found at concentrations below 100 ng/L in anthropogenically impacted river waters (Li et al., 2022). In our previous study during the 2020/2021 winter season (Haenelt et al., 2023), the median SMX concentration at Site 3 was 8.6 ng/L and therefore lower than during the summer season. Lower antibiotic concentrations in the effluent during the winter season could be explained by dilution effects due to precipitation in the WWTP catchment (Hong et al., 2018). Taking the SMX concentration as an indicator of the proportion of wastewater in the river, WWTP effluent would account for nearly 90% of the river water at Site 4 in the summer season of 2022. This value is similar to the value of 75% estimated on the basis of the flow rates provided by the State Office for Flood Protection and Water Management Saxony-Anhalt.^2^ Downstream of the discharge point, SMX concentration decreased. However, the decrease was not statistically significant and the decline rate of SMX concentration (11% from Site 3 to Site 6) was lower than during the winter season (73%), presumably because of high water evaporation together with the lack of precipitation. SDZ and SMZ concentrations did not exceed 1.3 ng/L at any of the sampling sites (Figure 1A). SMX and SMZ concentrations at Site 4 are consistent with a previous study conducted on the Holtemme (Tousova et al., 2017). There, samples were taken approximately 1 km downstream from our Site 4, the mean SMX concentration was 8 ng/L and SMZ concentrations were below the limit of quantification. SDZ concentrations were previously not quantified.

During the hot and dry summer season in 2022, water depth was on average below 10 cm in the Holtemme. Water temperature upstream of the WWTP varied between 13 and 16°C, while wastewater discharge at Site 3 (21°C) resulted in an elevated water temperature of 19°C at Site 4. Downstream at Site 5 (17°C), water temperature increased further, peaking at 22°C at Site 6. The pH in the river changed from pH 7.3 at Site 1 to pH 8.8 at Site 6. Nitrate concentrations were 1.2 mg/L upstream of the WWTP, 4 mg/L in the wastewater effluent, and 2.8 mg/L downstream at Site 5. These values are in the lower range of previously reported nitrate concentrations along the river stretch (Brase et al., 2018). Total phosphorous showed a similar pattern with low concentrations of 0.02 mg/L at Site 1 and an increase to 0.1 mg/L at Site 5 after WWTP discharge of 1 mg/L (Figure 1B).

### Bacterial community structure

**FIGURE 2:**
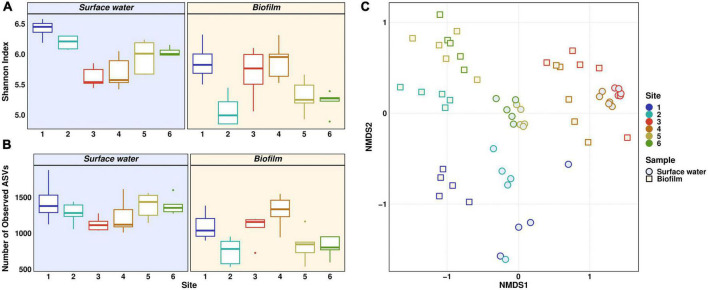
Microbial community structure and diversity based on 16S rRNA gene amplicon sequencing. Alpha diversity is given using Shannon measurement **(A)** and Number of observed ASVs **(B)**. Beta diversity is displayed as NMDS plot of Bray-Curtis-dissimilarities **(C)**. Distinct colors represent different sites. Light yellow background **(A,B)** and squares **(C)** represent biofilm samples, light blue background **(A,B)** and circles **(C)** represent surface water samples.

The bacterial community structures of planktonic and biofilm samples are reported individually, using Shannon index and species richness based on the number of observed Amplicon Sequence Variants (ASVs) to assess bacterial diversity (Figure 2). In planktonic samples, both indices showed the same trend. Highest median values of 6.4 and 1,379 were found at Site 1, whereas lowest values were found at Site 3 (median 5.5 and 1,113). The decrease in Shannon index from Site 1 and 2 to Site 3 was statistically significant (Dunn’s test: *p* ≤ 0.01 for Site 1, *p* ≤ 0.05 for Site 2). Downstream of the WWTP, both indices increased steadily, reaching values of 6 and 1,354 at Site 6. The steady increase of both indices downstream the discharge point indicates a recovery of the riverine bacterial community, which was demonstrated in our previous winter monitoring study on the Holtemme using a microbiome recovery model (Haenelt et al., 2023). In contrast to planktonic samples, both indices decreased in the biofilm samples from Site 1 (5.8 and 1,038) to Site 2 (5 and 782). Values at Site 3 (5.7 and 1,157) and nearby Site 4 (5.9 and 1,334) are similar to those at near-pristine Site 1, while further downstream the WWTP, both indices again decreased in a statistically significant manner (Dunn’s test: *p* ≤ 0.05) with medians of 5.3 and 803 at Site 6. Notably, the biofilm communities at Sites 1, 2, 5, and 6 showed significantly lower alpha diversity in comparison to the corresponding planktonic samples (Dunn’s test: *p* ≤ 0.05). The number of observed ASVs at Site 1 was the only exception, showing a non-significant difference. Conversely, polluted Sites 3 and 4 show much more uniform alpha diversity in both planktonic and biofilm-forming bacterial communities. We hypothesize that the increase in both indices in the surface water compartment downstream from the WWTP might be attributed to biases during rarefaction of the sequencing data. The high abundance of certain taxa in wastewater may have prevented detection of rare taxa, which were found further downstream in the river with higher evenness (Cameron et al., 2021).

The composition of bacterial communities along the river stretch was impacted by anthropogenic pollution. This is evident in the dissimilarities of beta diversity across different sites, as shown in the NMDS plot in Figure 2C. Influencing factors could be foremost the discharge of wastewater, flow rate, physicochemical parameters, and rectification (Luo et al., 2020). A clear trend could be observed with Sites 1 and 2 on one side and WWTP effluent and nearby Site 4 on the other side of the plot, whereas Sites 5 and 6 showed very similar community structure and are positioned in between the upstream and WWTP-impacted sites. Significantly distinct beta diversity was observed in both planktonic and biofilm-forming microbial communities across all sampling sites, except when comparing Site 3 to Site 4 (PERMANOVA: *p* ≤ 0.05). NMDS analysis also revealed a statistically significant separation between planktonic and biofilm-forming bacterial communities at all sampling sites (PERMANOVA: *p* ≤ 0.01). However, the change in community composition seemed to be driven more by the anthropogenic pollution rather than the type of compartment, i.e., surface water vs. biofilm.

**FIGURE 3:**
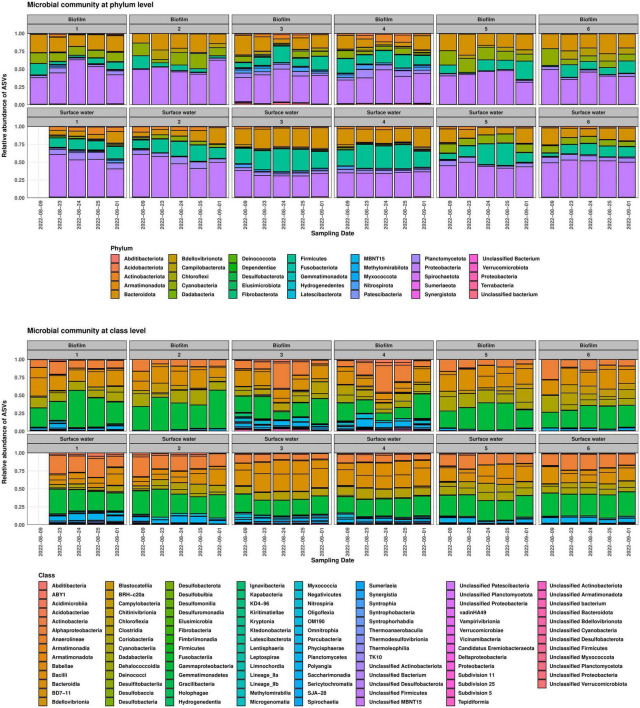
Microbial community structure at phylum **(top)** and class level **(bottom)** for each site. Sampling dates are displayed on the *x*-axis. Sample for Site 1, 8th August 2022 was removed before analysis due to low sampling depth.

The taxonomic compositions of the samples corresponded to the degree of anthropogenic influence along the river. The 50 most abundant phylotypes for each site and their corresponding relative abundances can be found in Supplementary Table 6. Biofilm and planktonic communities at Site 1 were dominated by members of the classes *Alphaproteobacteria*, *Gammaproteobacteria*, *Clostridia*, and *Bacilli*. In the biofilm samples, approximately 4% of the high-quality reads were derived from *Cyanobacteria*, whereas in the planktonic samples, *Cyanobacteria* were not among the 50 most abundant phylotypes (>0.26% of total read abundance). At the family level, bacteria typically exhibiting an aerobic, heterotrophic metabolism predominated in both compartments (e.g., *Chitinophagaceae*, *Comamonadaceae*, and *Xanthobacteraceae*). Noteworthy, we found an ASV affiliated with *Escherichia-Shigella* in every sample from the surface water compartment at Site 1 (average read abundance of 0.3%). Since identical or similar ASVs were not found in any of the other 55 samples from our campaign, it seems unlikely that they are sampling/laboratory contaminants in the planktonic samples from Site 1. While it remains to be elucidated to which genus the *Escherichia-Shigella* ASV belonged, and whether it was derived from an autochthonous population, the members of that genus could be a source of the occasionally detected *sul1*, *sul2*, and *intI1* genes at the site (see below). At Site 2 just downstream the city of Wernigerode, the communities of the surface water and biofilm compartments included several phylotypes often associated with warm-blooded animals (e.g., various *Ruminococcaceae*) in addition to those found at Site 1. The WWTP effluent at Site 3 was dominated by phylotypes of the family *Peptostreptococcaceae* and the order *Lactobacillales* in planktonic samples, while in biofilm samples most abundant phylotypes were associated with *Peptostreptococcaceae*, *Nitrospiraceae*, and *Rhodobacteraceae*. Downstream the discharge point, *Peptostreptococcaceae* remained a dominant family, but *Sphingomonadaceae* and *Xenococcaceae* were also detected with increasing abundance. *Arcobacteraceae* (phylum *Campilobacterota*) became highly abundant in biofilm samples (Figure 3). Members of this family have been identified as predominate hosts of *sul1*, *sul2*, *intI1*, and probably class 1 integrons in sewage-impacted water (Hultman et al., 2018; Knecht et al., 2023). Overall, the relative abundance of families with known hosts of integrons slightly decreased from 17% in both biofilm and surface water compartment at Site 1 to an average relative abundance of 12% downstream the WWTP, thus potential hosts for ARGs were already present upstream the WWTP in an area with little anthropogenic impact. Some of these families such as *Aeromonadaceae* and *Rhodocyclaceae* have been implicated as prevalent carriers of the indicator genes in and downstream of wastewater treatment systems (Zhang et al., 2018; Lo et al., 2022; Knecht et al., 2023).

**FIGURE 4:**
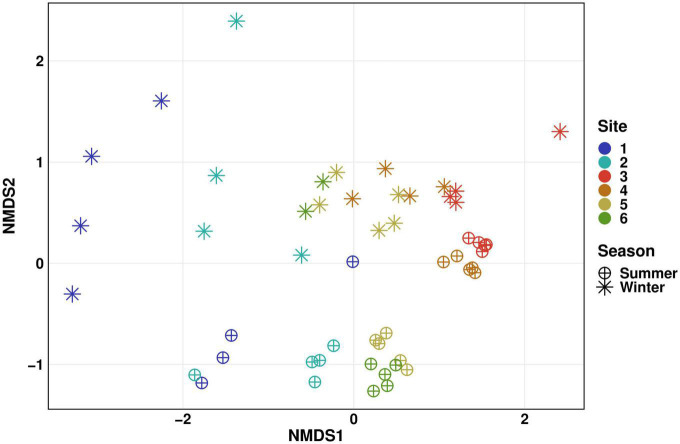
Seasonal comparison (winter vs. summer) of microbial community diversity in surface water displayed as NMDS plot of Bray-Curtis-dissimilarities. Distinct colors represent different sites. Stars represent samples taken during an earlier monitoring study in the winter season 2020/2021 (Figure 2C, Water), circles represent samples taken in this study in the summer season 2022 (Haenelt et al., 2023). Differences were statistically different between both seasons for all sampling sites, with Sites 3, 4, and 5 showing higher significance levels (PERMANOVA: *p* ≤ 0.01) than Sites 1, 2, and 6 (PERMANOVA: *p* ≤ 0.05).

Differences in beta diversity of planktonic communities from this sampling campaign and those analyzed in our study of the winter season 2020/2021 (Haenelt et al., 2023) are shown in Figure 4. Bacterial communities in summer and winter season were distinct, with statistically significant differences at all sampling sites (PERMANOVA: *p* ≤ 0.05). Summer communities cluster more than those acquired in winter season, which is most likely related to the shorter sampling period. This becomes evident in the spatial arrangement of data points in the NMDS plot depending on the sampling date in the winter season, where samples were collected over approximately 3 months. The summer sampling campaign lasted only about 1 month. In contrast to upstream sites, the bacterial community in the WWTP effluent were more stable over time and in between seasons, as data points are located close to each other in the plot. As WWTP performances are often stable over time, one would expect the bacterial community structure to be distributed more closely around an average, as previously shown by Wang et al. (2021). At downstream Sites 4, 5, and 6, site-specific distances are much smaller than the time-specific differences. These findings are consistent with a monitoring study conducted on a river in southern Germany (Reichert et al., 2021), illustrating the substantial impact of seasonality on bacterial community structure and diversity.

### ARG abundance

**FIGURE 5:**
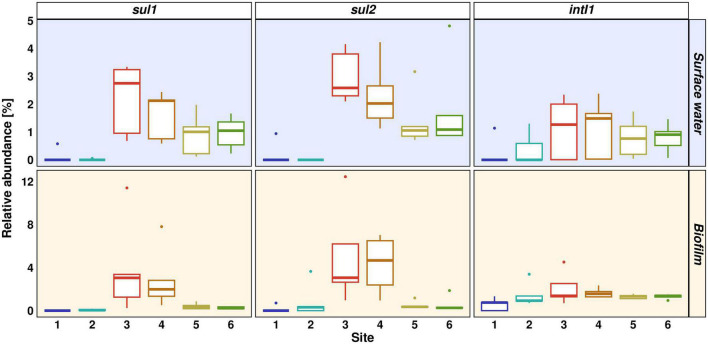
Abundance of *sul1*, *sul2*, and *intI1* relative to 16S rRNA gene. Distinct colors represent different sites. Light yellow background represents biofilm samples, light blue background represents surface water samples.

The anthropogenic pollution pattern in the Holtemme is evident from the prevalence of *sul1*, *sul2*, *intI1*, and the 16S rRNA gene in planktonic samples. All four genes showed a similar pattern with low CN or CN below limit of quantification at Sites 1 and 2, highest CN in wastewater and decreasing CN downstream of the WWTP (Supplementary Figure 3). The increase in absolute abundance was statistically significant for *sul1*, *sul2* and 16S rRNA gene from upstream Sites 1 and 2 to heavily wastewater-impacted Sites 3 and 4 (Dunn’s test: *p* ≤ 0.01). The median CN of 16S rRNA gene at Site 6 was only 16% of the CN at Site 4. Similar declines were observed for *sul1*, *sul2*, and *intI1* (12, 10, and 6%, respectively). The absolute CN of the 16S rRNA gene was higher at the wastewater discharge point (median 4.1 × 10^7^ CN/100 mL) than in the river water with a median of 4.3 × 10^6^ CN/100 mL at Site 5. In contrast to our previous monitoring study in the winter season 2020/2021, the absolute abundance of all four target genes in summer season declined more rapidly from Site 4 to 5. This observation is of special interest, as we would have expected the opposite, a faster decrease in the absolute abundance during winter season, due to dilution effects. Absolute abundance for biofilm samples is not depicted because a normalization to CN/sample does not allow for direct comparison between different sampling sites. In the summer season, relative abundance against 16S rRNA gene showed a similar pattern for all three target genes *sul1*, *sul2*, and *intI1* in planktonic samples, with the highest relative abundance at Site 3 (median 2.8, 2.6, and 1.3%, respectively) and decreasing relative abundance from Site 4 to 5 (Figure 5). Wastewater discharge at Site 3 resulted in significantly increased relative abundances of *sul1* and *sul2* when compared to both upstream sites (Dunn’s test: *p* ≤ 0.05). The decrease in absolute and relative abundance downstream the WWTP is most likely attributed to dilution effects, but additional removal mechanisms like sedimentation, cell death by predation or inactivation by UV light might also occur (Lee et al., 2021).

Although both, the relative and absolute abundances of these genes are decreasing, the median values remain largely unchanged 8 km (Site 5) and 13 km (Site 6) downstream the WWTP. These observations show that ARGs can be persistent in the aquatic environment even far downstream the point source of pollution (>10 km). Sabri et al. (2020) measured the abundance of several ARGs in a Dutch river and absolute abundances of *sul1*, *sul2*, and *intI1* gene copy numbers remained constant over 20 km of the river stretch regardless of the season. These findings differ from our previous monitoring study in winter season 2020/2021, where absolute abundances of *sul1* and *sul2* decreased to copy numbers below the limit of quantification at Site 6, 13 km downstream the WWTP. Potential causes for differing observations could be the fraction of wastewater discharge, and the amount of precipitation and additional inflow from tributaries depending on the season. In addition, the absolute abundance of the 16S rRNA and sulfonamide resistance genes was significantly higher in the WWTP effluent and at all sampling sites downstream the WWTP in the summer compared to the winter season (Dunn’s test: *p* ≤ 0.05). This increase is most likely a consequence of higher abundance of microorganisms in the WWTP during summer season. The relative abundance of *sul1* and *sul2* was also higher in the summer season at Site 3 and downstream the WWTP. For *sul2* the difference was statistically significant (Dunn’s test: *p* ≤ 0.05). We estimate an increase in the proportion of WWTP effluent at Site 4 from ∼27% in winter to about 75% in the summer season (see above). Therefore, seasonal differences like the increased proportion of wastewater and thus ARG-carrying wastewater-derived microorganisms in the river increased the abundance of sulfonamide resistance genes. In the face of climate change with less average precipitation in summer seasons in Europe, one must assume that the proportion of wastewater in rivers will increase even further (European Environment Agency, 2021), leading to an increase of ARGs in rivers.

In biofilm samples, similar relative abundances were observed for *sul1* and *sul2*, however, at higher relative abundance at Site 3 (both genes at 3%) and Site 4 (2 and 4.7%) and a more rapid drop from Site 4 to Site 5 (both 0.3%). The relative abundance of both genes was significantly increased at Site 3 and 4 when compared to the pristine Site 1 (Dunn’s test: *p* ≤ 0.01). The elevated relative abundance in vicinity to the discharge point indicates that ARG-carrying microorganisms were mainly accumulated in biofilm directly at the WWTP, whereas those remaining in surface waters over longer distance did not or only barely enter biofilms at a later stage. Similar findings were reported by Guo et al. (2018), where the authors found higher ARG abundances and detection frequencies in biofilm compared to planktonic samples. The accumulation of antibiotics in the biofilm compartment, in combination with dense microbial growth and accumulation of mobile genetic elements might render biofilms an important reservoir for ARGs downstream WWTPs (Gillings et al., 2009). However, the spatially restricted occurrence of sulfonamide resistance genes *sul1* and *sul2* in biofilms in close vicinity to the discharge point suggests that this was not the case in our study.

In contrast to the sulfonamide resistance genes, *intI1* was detectable upstream and downstream of the WWTP without significant differences in the relative abundance between sampling sites. However, wastewater discharge increased its relative abundance from a median of 0.9% at Site 2 to 1.5% at Site 4. All anthropogenically impacted ecosystems have become polluted with the clinical class 1 integron in recent years (Gillings, 2018). For example, Cacace et al. (2019) conducted a study on the abundance of nine ARGs and the *intI1* gene in 16 WWTP effluents from 10 European countries. Similar to the biofilm samples we studied, they found a background level of *intI1* resistance already present upstream of the WWTP, which further increased when wastewater entered the river. Possible reasons for the occurrence upstream of a WWTP could be anthropogenic impact from the city of Wernigerode or leisure activities at the river (Weitere et al., 2021). The prevalence of the *intI1* gene in biofilm throughout the river stretch is of concern if correlating with the abundance of class 1 integron, as the integron could potentially carry various ARGs as gene cassettes, and biofilms provide suitable conditions for horizontal gene transfer (Gillings et al., 2008; Chaturvedi et al., 2021).

During our monitoring study, we were limited in information regarding flow rates for all sampling sites, which would have been needed to calculate the loads of ARGs in CN/day as previously done by Lee et al. (2021). This would have further enabled us to determine the environmental fate of ARGs (e.g., dilution of wastewater in the river vs. additional removal mechanism) more precisely. In addition, single-cell approaches such as epicPCR or directGeneFISH (Moraru et al., 2010; Spencer et al., 2016) could be implemented in future studies to link the host phylotypes with the ARGs type and to better assess the risk of ARG transfer to human pathogens.

## Conclusion

The findings of this study emphasize the importance of spatiotemporal monitoring of anthropogenic pollution on both planktonic and biofilm-forming bacterial communities in aquatic ecosystems. Higher proportions of wastewater in the river increased the absolute and relative abundance of sulfonamide resistance genes in summer when compared with previously published data obtained during the winter season. Even though we observed a more rapid decline of absolute abundances after wastewater discharge in the summer season, we found that the indicator genes *sul1*, *sul2*, and *intI1* were persistent in river water and remained detectable even 13 km downstream of the pollution source, which was not the case in our previous study in the winter season. In light of climate change, where forecasts predict even hotter and drier summers and therefore greater urban discharge fractions in streams (European Environment Agency, 2021), this finding become of great concern. Moreover, resistance genes were found to be higher abundant in biofilms located near the WWTP in comparison to the surrounding water, suggesting the possible accumulation of antibiotic-resistant bacteria derived from wastewater in biofilms. Further research is needed to understand the underlying mechanisms responsible for the accumulation of ARGs in biofilms and to gain better insights into the influence of seasonal variations on bacterial community alteration and ARG persistence in aquatic environments. It is already certain that higher proportions of wastewater in rivers and the resulting high occurrence of wastewater-derived microorganisms lead to the increased abundance and persistence of ARGs in the aquatic environment.

## Data availability statement

The datasets presented in this study can be found in online repositories. The names of the repository/repositories and accession number(s) can be found below: https://www.ncbi.nlm.nih.gov/, PRJNA962299.

## Author contributions

SH, NM, JM, and H-HR conceived the study and the experimental design. SH performed the sampling, sample treatment, and data analysis and wrote the manuscript with contributions from NM and JM. NM and SH supervised during the whole study duration. All authors contributed to the manuscript revision and read and approved the submitted version.

## Footnotes

1
https://www.wahb.eu/

2
https://hochwasservorhersage.sachsen-anhalt.de/messwerte/wasserstand
