# Supplementary material for: Antibiotic resistance indicator genes in biofilm and planktonic microbial communities after wastewater discharge
Source: Front Microbiol. 2023 Sep 5;14:1252870. doi: 10.3389/fmicb.2023.1252870 (PMC10507703; doi:10.3389/fmicb.2023.1252870)
Supplement: Supplementary file 2 [file Data_Sheet_1.pdf]

Supplementary Material

1 Supplementary Tables

Table S1. Overview of primers used in this study.

Gene	Primers*	Sequence (5'-3')	NCBI Ref.Seq.	Amplicon size (bp)	Reference
16S	27F	AGAGTTTGATCMTGGCTCAG	-	1465	Lane D. J., 1991
	1492R	CGGTTACCTTGTTACGACTT			Turner et al., 1999
	519qF	CCAGCAGCCGCGGTAATAC		410	
	909qR	CCGTCAATTCCTTTRAGTTT			
	341F-TS	ACACTCTTTCCCTACACGACGCTCTTCCGATCT|CCTACGGGNGGCWGCAG	-	194	
	518R-TS	GACTGGAGTTCAGACCTGTGCTCTTCCGATCT|WTTACCGCGGCTGCTGG	-		
sul1	sul1qF	TGTCGAACCTTCAAAAGCTG	WP_000259031.1	113	Wang et al., 2014
	sul1qR	TGGACCCAGATCCTTTACAG			
sul2	sul2qF	ATCTGCCAAACTCGTCGTTA	WP_001043260.1	89	Wang et al., 2014
	sul2qR	CAATGTGATCCATGATGTCG			
intI1	intI1qF	CGAACGAGTGGCGGAGGGTG	WP_000845048.1	312	Gillings et al., 2015
	intI1qR	TACCCGAGAGCTTGGCACCCA			

*q in primer name indicates the use in qPCR

Table S2. Statistical significance of differences in alpha diversity measured by Shannon Index (top) and Number of Observed ASV (bottom). The significance of differences between sampling sites was evaluated for surface water (left) and biofilm compartment (right) using Dunn's test. Additionally, within each sampling site, the significance of differences comparing surface water and biofilm compartment, was calculated (middle). Significant differences between sites are represented by asterisks (ns > 0.05, * ≤ 0.05, ** ≤ 0.01).

**Shannon Index**														
**Surface water**							**Surface water vs biofilm**	**Biofilm**						
1	2	3	4	5	6	Site		Site	1	2	3	4	5	6
	ns	**	**	ns	ns	1	*	1		*	ns	ns	ns	*
		*	*	ns	ns	2	**	2			*	*	ns	ns
			ns	ns	ns	3	ns	3				ns	ns	ns
				ns	ns	4	ns	4					ns	*
					ns	5	**	5						ns
						6	**	6						
**Number of Observed ASVs**														
**Surface water**							**Surface water vs biofilm**	**Biofilm**						
1	2	3	4	5	6	Site		Site	1	2	3	4	5	6
	ns	ns	ns	ns	ns	1	ns	1		ns	ns	ns	ns	ns
		ns	ns	ns	ns	2	**	2			ns	*	ns	ns
			ns	ns	ns	3	ns	3				ns	ns	ns
				ns	ns	4	ns	4					*	*
					ns	5	*	5						ns
						6	**	6						

Table S3. Statistical significance of differences in beta diversity, presented by non-metric multidimensional scaling (NMDS) of Bray-Curtis dissimilarities. The significance of differences between sampling sites was evaluated for surface water (left) and biofilm compartment (right) applying PERMANOVA. Additionally, within each sampling site, the significance of differences comparing surface water and biofilm compartment, was calculated (middle). Significant differences between sites are represented by asterisks (ns > 0.05, * ≤ 0.05, ** ≤ 0.01).

**Surface water**							**Surface water vs biofilm**	**Biofilm**						
1	2	3	4	5	6	Site		Site	1	2	3	4	5	6
	*	**	**	**	**	1	**	1		**	**	**	**	**
		**	**	**	**	2	**	2			**	**	**	**
			ns	**	**	3	**	3				ns	**	**
				**	**	4	**	4					**	**
					*	5	**	5						*
						6	**	6						

Table S4. Statistical significance of differences in the relative abundance of *sul1* (top), *sul2* (middle), and *intI1* (bottom). The significance of differences between sampling sites was evaluated for surface water (left) and biofilm compartment (right) using Dunn's test. Additionally, within each sampling site, the significance of differences comparing surface water and biofilm compartment, was calculated (middle). Significant differences between sites are represented by asterisks (ns > 0.05, * ≤ 0.05, ** ≤ 0.01, *** ≤ 0.001).

sul1														
**Surface water**							**Surface water vs biofilm**	**Biofilm**						
1	2	3	4	5	6	Site		Site	1	2	3	4	5	6
	ns	**	*	ns	ns	1	ns	1		ns	***	***	ns	ns
		**	*	ns	ns	2	ns	2			*	*	ns	ns
			ns	ns	ns	3	ns	3				ns	ns	ns
				ns	ns	4	ns	4					ns	ns
					ns	5	ns	5						ns
						6	*	6						

sul2														
**Surface water**							**Surface water vs biofilm**	**Biofilm**						
1	2	3	4	5	6	Site		Site	1	2	3	4	5	6
	ns	*	*	ns	ns	1	ns	1		ns	**	**	ns	ns
		**	**	ns	*	2	ns	2			ns	ns	ns	ns
			ns	ns	ns	3	ns	3				ns	ns	ns
				ns	ns	4	ns	4					ns	ns
					ns	5	*	5						ns
						6	ns	6						

intI1														
**Surface water**							**Surface water vs biofilm**	**Biofilm**						
1	2	3	4	5	6	Site		Site	1	2	3	4	5	6
	ns	ns	ns	ns	ns	1	ns	1		ns	ns	ns	ns	ns
		ns	ns	ns	ns	2	*	2			ns	ns	ns	ns
			ns	ns	ns	3	ns	3				ns	ns	ns
				ns	ns	4	ns	4					ns	ns
					ns	5	ns	5						ns
						6	ns	6						

Table S5. Statistical significance of differences in the relative and absolute abundance of *sul1*, *sul2*, *intI1*, and 16S rRNA gene comparing data obtained during this summer season with data from a previous publication conducted during winter season (Haenelt et al., 2023). The significance of differences within each sampling site was calculated using Dunn's test. Significant differences between sites are represented by asterisks (ns > 0.05, * ≤ 0.05, ** ≤ 0.01, *** ≤ 0.001).

	Relative abundance			Absolute abundance			
**Site**	*sul1*	*sul2*	*intI1*	16S	*sul1*	*sul2*	*intI1*
1	ns	ns	ns	ns	ns	ns	*
2	ns	ns	ns	ns	ns	ns	ns
3	*	**	ns	**	**	**	ns
4	ns	*	ns	**	**	**	ns
5	ns	*	ns	*	**	**	ns
6	ns	***	*	**	**	***	ns

2 Supplementary Figures

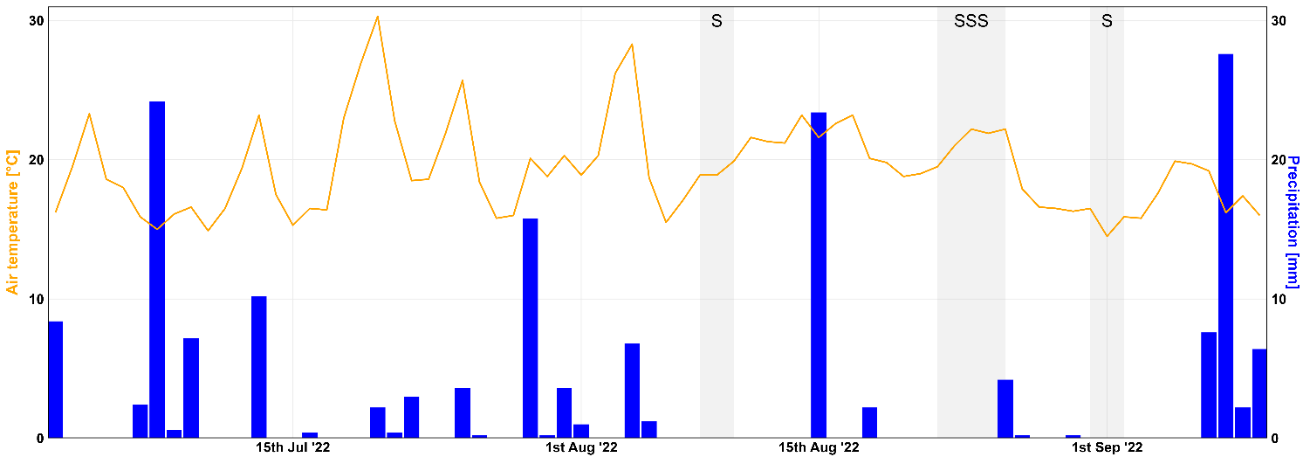

Figure S1. Air temperature (yellow line) and precipitation (blue bar) in Wernigerode, Germany from 1st July to 15th September 2022. Sampling events are highlighted in grey and labelled with an “S”. Data was received from the German weather monitoring station 5490 (https://opendata.dwd.de/).

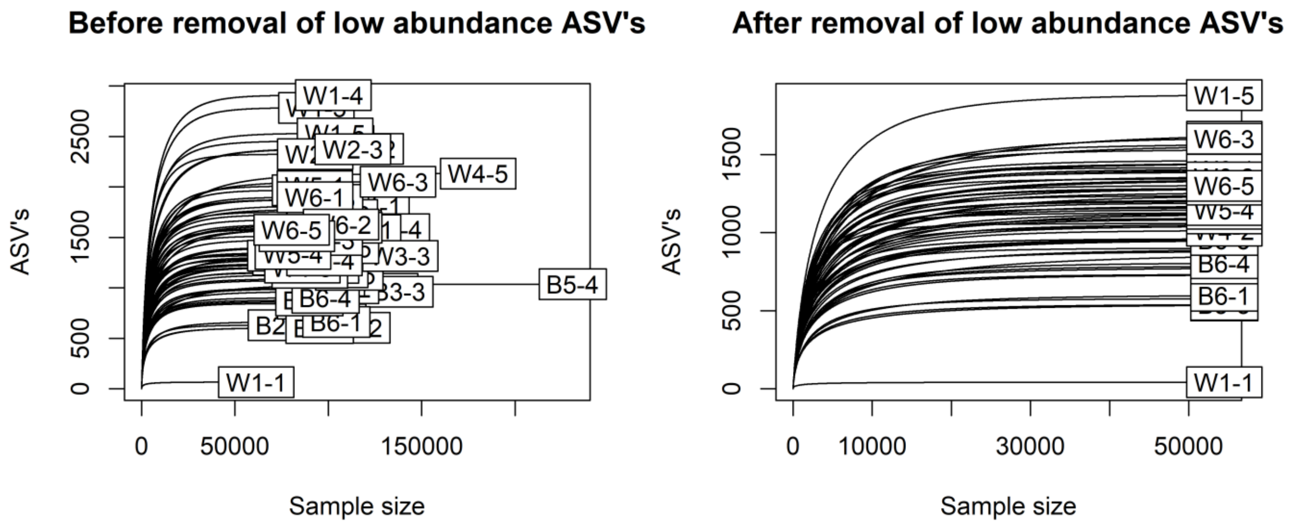

Figure S2. Rarefaction curves for 16S rRNA gene sequencing before (left) and after (right) removal of low abundance ASV’s.

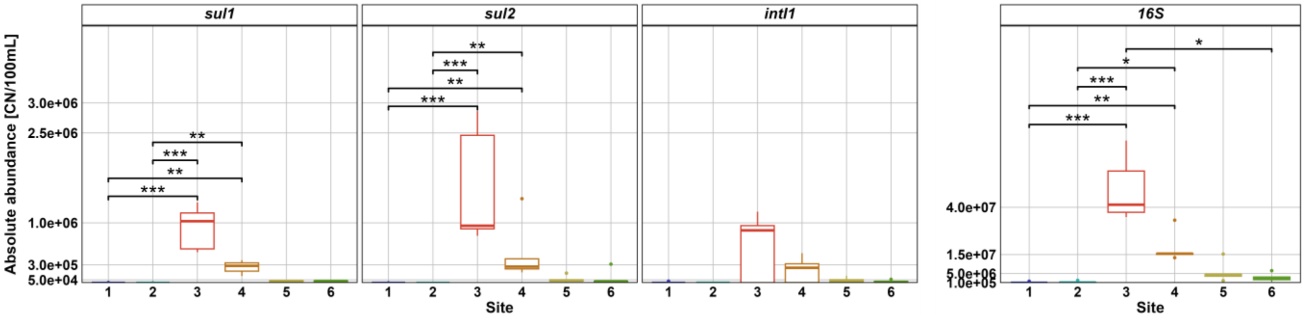

Figure S3. Absolute abundance of *sul1*, *sul2*, *intI1* and 16S rRNA gene in planktonic samples, determined by quantitative real-time PCR. Significant differences between sites are represented by asterisks (Dunn's test, * ≤ 0.05, ** ≤ 0.01, *** ≤ 0.001). Number of replicates (n) = 5.

3 References

Gillings, M. R., Gaze, W. H., Pruden, A., Smalla, K., Tiedje, J. M., and Zhu, Y.-G. (2015). Using the class 1 integron-integrase gene as a proxy for anthropogenic pollution. *ISME J* 9, 1269–1279. doi: 10.1038/ismej.2014.226

Haenelt, S., Wang, G., Kasmanas, J. C., Musat, F., Richnow, H. H., da Rocha, U. N., et al. (2023). The fate of sulfonamide resistance genes and anthropogenic pollution marker intI1 after discharge of wastewater into a pristine river stream. *Front Microbiol* 14, 1058350. doi: 10.3389/fmicb.2023.1058350

Lane D. J. (1991). 16S/23R rRNA sequencing. *Nucleic acid techniques in bacterial systematics.*, 115–175.

Turner, S., Pryer, K. M., Miao, V. P., and Palmer, J. D. (1999). Investigating deep phylogenetic relationships among cyanobacteria and plastids by small subunit rRNA sequence analysis. *J Eukaryotic Microbiology* 46, 327–338. doi: 10.1111/j.1550-7408.1999.tb04612.x

Wang, N., Yang, X., Jiao, S., Zhang, J., Ye, B., and Gao, S. (2014). Sulfonamide-resistant bacteria and their resistance genes in soils fertilized with manures from Jiangsu Province, Southeastern China. *PLoS One* 9, e112626. doi: 10.1371/journal.pone.0112626
